# The Spectrum of Erythema Migrans in Early Lyme Disease: Can We Improve Its Recognition?

**DOI:** 10.7759/cureus.30673

**Published:** 2022-10-25

**Authors:** Anna M Schotthoefer, Clayton B Green, George Dempsey, Elizabeth J Horn

**Affiliations:** 1 Marshfield Clinic Research Institute, Marshfield Clinic Health System, Marshfield, USA; 2 Dermatology, University of Rochester Medical Center, Rochester, USA; 3 Family Medicine, East Hampton Family Medicine, East Hampton, USA; 4 Biobank, Lyme Disease Biobank, Portland, USA

**Keywords:** tick-borne disease, skin infection, skin lesion, erythema migrans, lyme disease

## Abstract

Background and objective

Diagnosis of early Lyme disease (LD) often relies on clinical recognition of the skin lesion, erythema migrans (EM), a diagnostic sign of disease when laboratory testing is insensitive. Because EM can present in morphologically distinct forms, its recognition by clinicians can be challenging. This study aimed to characterize the clinical spectrum of lesions in patients presenting with suspected early LD in an ambulatory care setting to identify features that might help clinicians to be better prepared to recognize EM lesions.

Methods

Images of lesions from 69 participants suspected to have early LD were retrospectively evaluated by a dermatologist and a family practitioner with expertise in early LD. Reviewers made determinations on the diagnoses and morphological features of lesions. Agreement between reviewers and associations among lesion types and participant demographics, symptomology, and laboratory evidence of infection were examined using the kappa statistic and contingency tables, respectively.

Results

Challenges in diagnosing EM were evident in our study: initial concordance between reviewers was moderate [kappa statistic (95% CI): 0.45 (0.245 - 0.657)]. The final classification included 35 lesions (51%) that were agreed to be EM; 23 lesions (30%) were considered to be possible early EM or tick bite reactions, and 11 (16%) were thought not to be EM, but rather other diagnoses, including ringworm, allergic contact dermatitis, and mosquito bites. Only two lesions (6%) were observed with a classic bull’s eye or ring-within-a-ring pattern. Most EM lesions were uniform (51%), pink (74%), oval lesions (63%), with well-demarcated borders (92%). Early EM or tick bite reactions were typically <5 cm in size (74%), red (52%), round lesions (61%), with a punctum present (100%). Lesions thought not to be EM also tended to be pink or red (64%), round (55%), or uniform (45%) lesions, but also had raised (25%) or irregular borders (33%), which were not commonly observed in the reviewer-classified EM or tick bite reaction lesions. Participants with lesions classified as EM reported that they had the lesions for more days (p = 0.043) and reported more symptoms (p = 0.017) than participants with other lesions. Only 14 (20%) participants overall had positive laboratory evidence for LD; these included 13 (37%) of the participants with EM-classified lesions.

Conclusions

EM commonly occurs in forms that are not the classic bull’s eye. Patients often present with lesions that may represent the very early stage of EM or tick bite reactions, and most patients will test negative on currently available laboratory tests, challenging clinicians in making an LD diagnosis or treatment decisions. Additional studies to further characterize the morphological features of EM and how variation in skin lesions may be perceived among clinicians would be helpful for developing guidelines on improving clinician recognition of EM.

## Introduction

Lyme disease (LD) in the United States is caused by infection with the spirochete bacteria, *Borrelia burgdorferi (B. burgdorferi) *and *Borrelia mayonii* [1,2], transmitted to people by the bite of an infected *Ixodes* tick. It is the most prevalent vector-borne disease in the United States, with most recent estimates suggesting that as many as 476,000 people may be diagnosed with the disease every year [1,3]. Areas of high incidence are concentrated in the Northeast, Mid-Atlantic, Upper Midwest [1], and some areas of California [4]. The persistent rise in incidence and the geographic expansion of the disease make it a growing public health problem.

A skin lesion known as erythema migrans (EM) is often the earliest sign of LD and is estimated to appear in more than 70% of early cases [1]. The primary lesion forms at the site of the infected tick bite within a few days or up to one month following the bite. As LD progresses, secondary EM lesions may form at other locations on the body. Patients typically develop systemic, non-specific flu-like symptoms such as headache, fatigue, fever, chills, myalgia, and joint pain during the early stage of the disease. If not treated promptly, infections can develop into more complicated, and often long-term manifestations involving the musculoskeletal, neurologic, and cardiac systems. Prompt diagnosis and treatment are the most effective ways to prevent severe illness and complications associated with LD [5-8]. However, laboratory diagnosis has significant limitations, as it relies primarily on serologic testing, which is insensitive in patients with EM and other early manifestations [9,10]. As such, early LD diagnosis tends to be a clinical diagnosis, relying on the abilities of clinicians to recognize EM in LD patients that present with the lesion, and in fact, national guidelines recommend that patients presenting with EM be treated without testing, as an EM is a more sensitive sign of early LD than findings of serologic testing [6,11].

Nonetheless, recognizing EM can be challenging. The primary lesion generally begins as a red macule or papule at the site of an infected tick bite. As the lesion expands, it may develop a central clearing resembling a red ring, or ring-within-a-ring, often described as a “bull’s eye” or target [12,13]. These central clearing forms have come to be known as the classic EM and are often emphasized in clinician teaching materials and scientific review articles, but are not representative of all presentations of EM. Expanding homogeneous lesions without central clearing, or lesions with dusky or more intense central erythema have also been described as commonly occurring [7,14,15], and atypical forms that have a vesiculobullous or hemorrhagic center may also occur [16]. Clinicians may not be aware of all existing variations, such that some LD patients with EM may not be immediately recognized and promptly diagnosed and treated [17-20]. Hence, further improvements in terms of clinician awareness and recognition of EM are needed.

The Lyme Disease Biobank (LDB) was created in 2014 to facilitate research in LD and other tick-borne infections (TBI). It collects whole blood, serum, and urine, from both prospectively enrolled participants exhibiting signs and symptoms of early LD and endemic controls. Data on presenting symptomology, images of skin lesions, and laboratory testing results for LD and other TBIs are also available for all participants [21]. We leveraged this unique dataset from participants enrolled in the Upper Midwest to evaluate and characterize the physical features and potential variation of EM in relation to other clinical features in this endemic LD region. We believe such characterizations may aid in designing guidelines on how to diagnose the full spectrum of EM.

## Materials and methods

The LDB participants enrolled at the Marshfield Clinic Health System (MCHS) in Wisconsin during 2016-2019 were eligible for enrollment in this study (Marshfield Clinic Research Institute IRB protocol SCH20216; Advarra IRB protocol Pro00012408). MCHS is an integrated healthcare system serving about 328,000 patients residing in central and northern Wisconsin. The region is a high-risk LD area as defined by the CDC, with annual incidence rates exceeding 10 per 100,000 persons [1]. Participants were recruited primarily from clinic locations in the cities of Marshfield, Wausau, Weston, Minocqua, Eau Claire, and Lake Hallie. Individuals who were aged 10 years or older were eligible, although the majority of participants in the LDB were adults (>18 years old).

Presentation to an MCHS provider with a cutaneous lesion believed to be an EM lesion (>5 cm) or an expanding EM (≤5 cm) was required for inclusion in the current study. At the time of enrollment, lesions were digitally photographed, measured, and described in terms of body location, appearance, and duration. Participants who did not have images or did not have images of sufficient quality for evaluation were excluded.

Participants completed questionnaires about other signs and symptoms of early LD, any known tick bites, their medical history, including any previous diagnoses for LD and TBIs, and their current medications. Blood was drawn from all participants for acute-phase LD testing. Donation of a second, convalescent blood draw for LD testing 60-90 days later was optional for participation in the LDB [21].

All laboratory testing on the LDB samples collected at MCHS was performed by Mayo Clinic Laboratories (Rochester, MN) and Stony Brook University (Stony Brook, NY). Blood was tested by real-time PCR for *B. burgdorferi* DNA and the other tick-borne pathogens: *Anaplasma phagocytophilum*, *Ehrlichia* species, and *Babesia microti* [22-24]. Serologic testing for detection of anti-*B. burgdorferi* antibodies used the standard two-tier testing (STTT) approach and included first-tier ELISAs and second-tier immunoblot testing for IgM and IgG [25]; the latter tests, however, were performed on all samples regardless of the first-tier results [21].

Interpretation of serologic testing results followed STTT guidelines [25]. Accordingly, the STTT result was considered positive evidence for early LD when at least one ELISA was positive or equivocal and the IgM and/or the IgG immunoblot test was positive in the acute-phase blood sample. Convalescent-phase blood samples were considered positive when at least one ELISA was positive or equivocal and the IgG immunoblot was positive, as it is recommended that a positive IgM immunoblot should not be used to support a diagnosis of active LD if signs and symptoms have been present for more than 30 days [25]. Participants were considered to have seroconverted if the IgG immunoblot test was positive at the convalescent visit after the STTT was negative at the acute visit.

To obtain expert opinions on the identities of the lesions for the present study, images of the suspected EM lesions were evaluated by a board-certified dermatologist (CG) and a board-certified family physician (GD). Both investigators have practiced medicine in LD-endemic regions for over a decade, with experience in diagnosing and treating early LD. They have served as investigators on the LDB and other LD clinical trials, though they were not involved in the enrollment of the LDB participants included in this study. The reviewers knew that the images came from LDB participants with suspected EM but were blind to other participant clinical data, including laboratory testing results. Reviewers initially examined the images blind to each other’s reviews and determined whether they thought the lesions were EM. If the reviewers thought the lesion was not an EM, or if they were uncertain, they recorded an alternate diagnosis. For this study, we did not require that the lesion was >5 cm to be considered an EM. The diagnoses recorded by reviewers fell into four possible categories: EM, possible or early EM, a tick bite reaction, or a lesion not thought to be EM with a different diagnosis.

To determine if there were morphological characteristics distinctive for EM lesions that would be useful for teaching clinicians how to recognize EM and differentiate it from other lesions, reviewers answered questions about the shape, color, evenness of color (homogeneity), pattern, and border characteristics of the lesions. The four possible choices for lesion patterns were ring-within-a-ring (which included the classic, bull’s eye or target-like EM), lesion with central darkness, lesion with central lightness or clearing, and uniform lesion, consisting of a homogeneous plaque without central darkness or clearing. Patterns not fitting into these categories were placed into the “other” category. Whether the lesions had a punctum or hemorrhagic puncture mark associated with an arthropod bite site was also noted.

Inter-reviewer agreement on the initial assignment of lesions was examined by using contingency tables and calculating the kappa statistic [26]. When the reviewers differed on their diagnoses, they worked towards a consensus on the lesion’s inclusion in one of the final four lesion categories. Inter-reviewer agreement on the morphological features of lesions was also examined. Features that were viewed differently were reevaluated and classified into an agreed-upon feature category.

Associations among the four reviewer-classified lesion categories and participant sex, presence of LD symptoms, laboratory evidence of LD, and the morphological features of lesions were examined by using contingency tables and calculating p-values using Fisher’s exact tests. The continuous variables, participant ages, size of lesions, and duration of lesions (days) were compared among lesion categories using analysis of variance. A p-value of 0.05 was used to assess significance. Analyses were conducted using SAS software, version 9.4 (SAS Institute, Inc., Cary, NC).

## Results

Of the 84 LDB participants who presented with a skin lesion at MCHS, 69 (82%) were included in the present study. Nine participants could not provide images, and six additional participants were excluded because their images were of poor quality, primarily because they were overexposed. Participants included 43 (62%) men and 26 (38%) women (Table 1). Only three (4%) participants were <18 years old, and all participants reported their race as White or non-Hispanic. Participants primarily presented with a single lesion (83%) (Table 1).

**Table 1:** Participant characteristics within each reviewer-classified lesion category Values are n (%) unless otherwise indicated *Not available for all participants; denominators were 26, 6, 10, 7, and 49 for erythema migrans, suspected early erythema migrans, tick bite reaction, other, and total, respectively EM: erythema migrans

Characteristic	EM (n = 35)	Suspected early EM (n = 7)	Tick bite reaction (n = 16)	Not-EM (n = 11)	Total (n = 69)
Median age (IQR) in years	62 (48 - 68)	74 (65 - 78)	57 (36 - 64)	67 (55 - 76)	63 (53 - 71)
Male	24 (69)	6 (86)	7 (44)	6 (55)	43 (62)
Female	11 (31)	1 (14)	9 (56)	5 (45)	26 (38)
Single lesion	26 (74)	7 (100)	15 (94)	9 (82)	57 (83)
Multiple lesions	9 (26)	0	1 (6)	2 (18)	12 (17)
Median (IQR, min, max) duration of lesion(s) in days*	5.5 (3 - 10, 1, 21)	2.5 (2 - 5, 1, 8)	2.5 (1 - 4, 1, 11)	4 (2 - 5, 2, 7)	4 (2 - 7, 1, 21)
Median (IQR, min, max) size of lesion(s) (cm)	9 (7 - 15, 4, 20)	4 (3 - 6, 3, 7)	3 (2.5 - 5.5, 1, 8)	6 (3 - 12, 2, 29)	7 (4 - 11, 1, 29)
Mode month (count) of presentation and enrollment	Jun (11)	May (4)	Apr (6)	May, Jul (3)	Jun (17)
Reported tick bite	18 (51)	6 (86)	14 (87)	5 (45)	43 (62)

**Figure 1:**
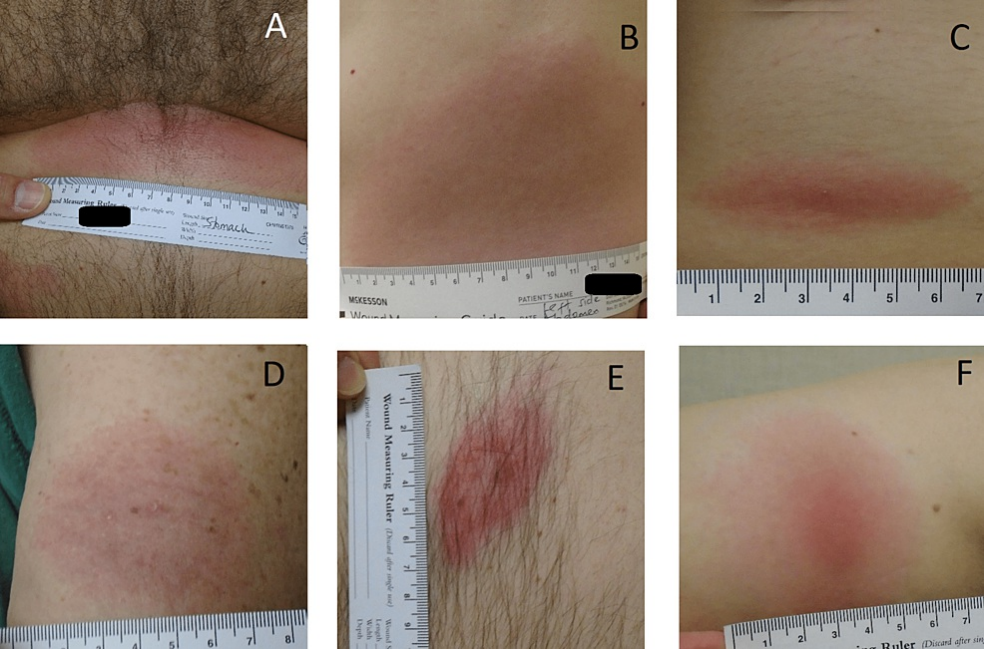
Images representing the variation in reviewer-classified EM lesions in our study (A-E) lesions with a uniform pattern; (F) lesion with a darkened center Participants in (A) and (B) had positive standard two-tier testing serologic evidence for LD EM: erythema migrans; LD: Lyme disease

**Figure 2:**
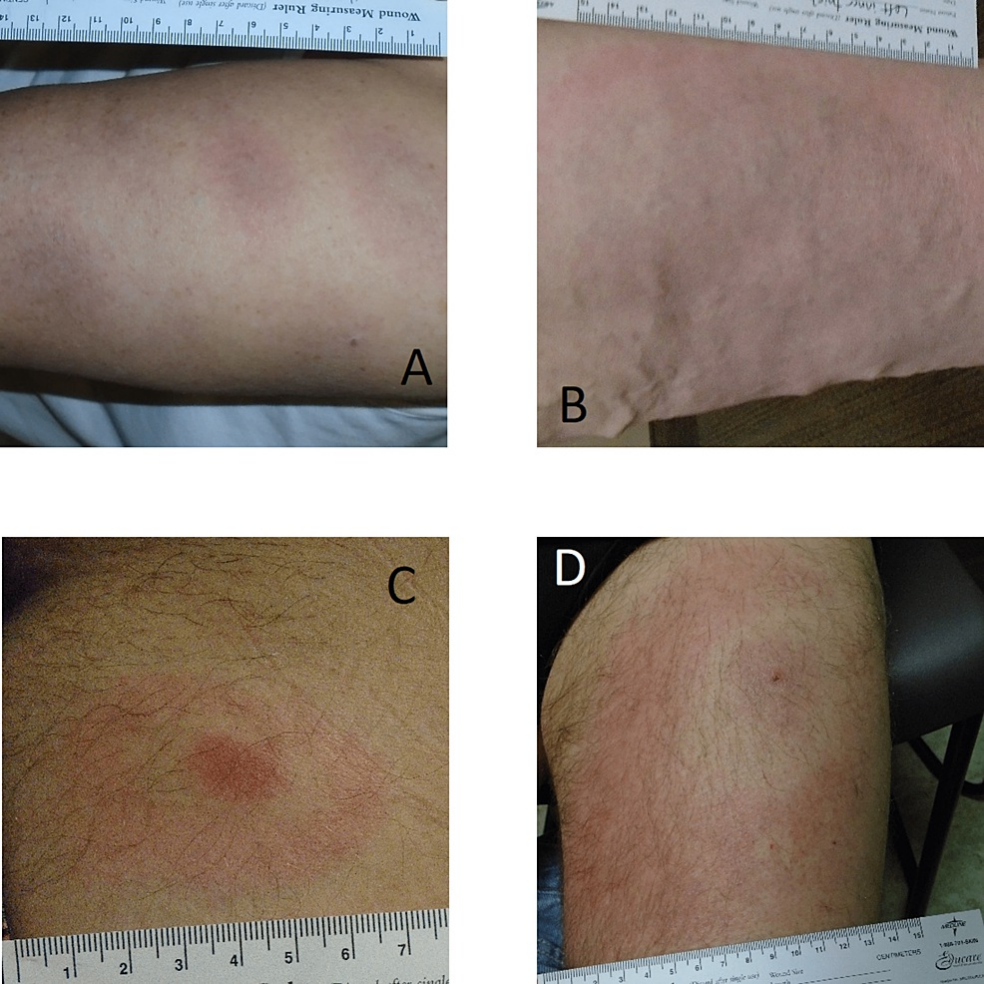
Additional images representing the variation in reviewer-classified EM lesions in our study (A) example of disseminated EM; (B) lesion with central clearing; (C) and (D) lesions with the classic, ring-within-a-ring, or bull’s eye, pattern. Participant in (A) had positive standard two-tier testing serologic evidence for LD and (C) was PCR-positive EM: erythema migrans; LD: Lyme disease; PCR: polymerase chain reaction

**Figure 3:**
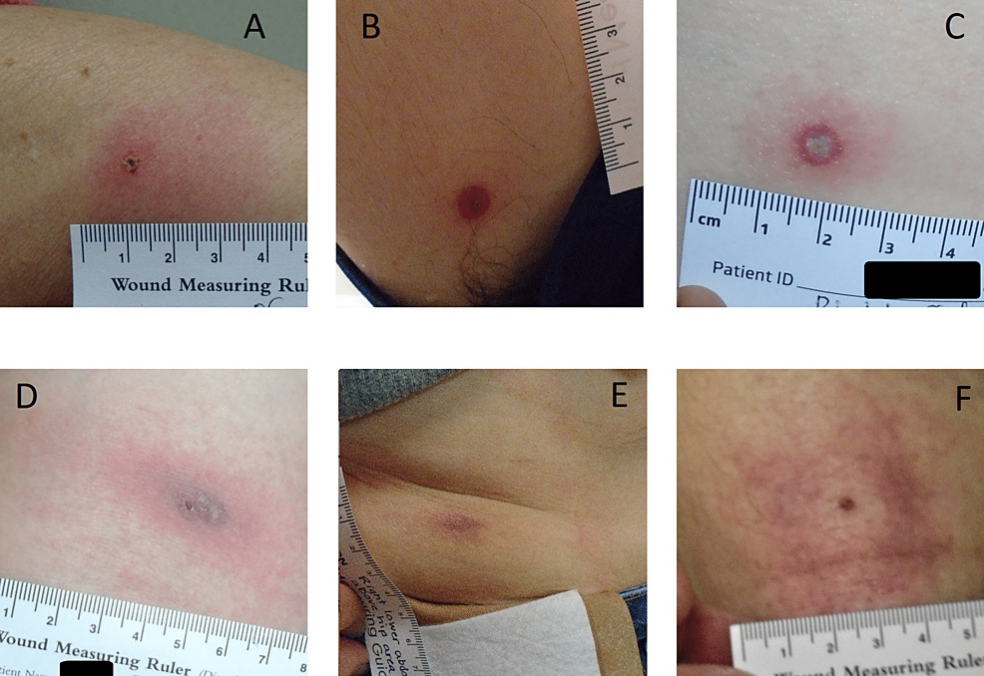
Lesions reviewer-classified as (A) possible early EM; (B-F) tick bite reactions Participant in (D) had positive serologic evidence for LD; (E) had bandage dermatitis overlapping the tick bite reaction; and (F) is an example of pruritus associated with tick bite reaction EM: erythema migrans; LD: Lyme disease

**Figure 4:**
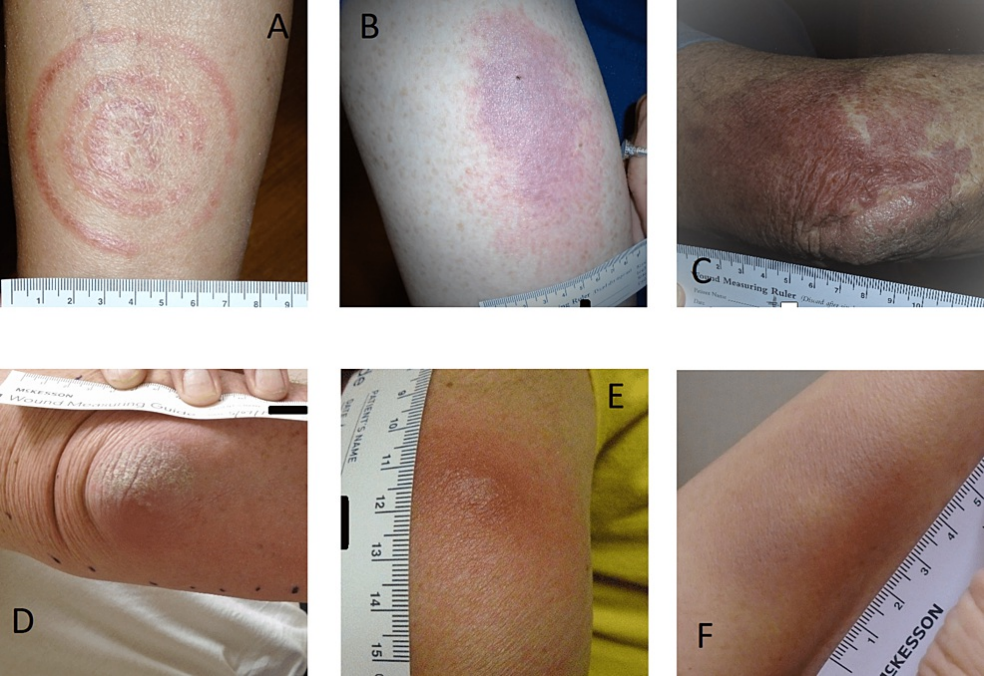
Lesions categorized into the not-EM lesion group, believed to have other diagnoses by reviewers (A) ringworm; (B) allergic contact dermatitis, possibly related to an arthropod bite or sting; (C) solar purpura; (D) olecranon bursitis with psoriatic patch; (E) mosquito or other arthropod bite; (F) hematoma or trauma EM: erythema migrans

Upon the initial evaluation of the skin lesions, reviewers agreed on the interpretation of 42 (61%) of 69 lesions; 28 (41%) of the lesions were agreed to be EM; two (3%) were thought to be possible early EM; 10 (14%) were identified as tick bite reactions; and two (3%) were agreed to be lesions of different diagnoses. There was moderate inter-reviewer agreement in this initial classification into the EM category versus all other categories [kappa statistic (95% confidence interval): 0.45 (0.245 - 0.657)]. Following a reevaluation of the 27 lesions that had initial discordant diagnoses, the final classification of lesions was as follows: 35 (51%) EM, seven (10%) possible early EM, 16 (23%) tick bite reactions, and 11 (16%) not-EM (Figures 1-4).

One lesion classified into the not-EM category had a definitive diagnosis of ringworm (Figure 4A); however, there was uncertainty regarding the diagnoses of the 10 other lesions in this category. Possible other diagnoses included allergic contact dermatitis, solar purpura, olecranon bursitis with a psoriatic patch on the elbow, hematoma or trauma, a prurigo nodule, herald patch of pityriasis rosea, and mosquito bites (Figure 4B-4E).

There was moderate inter-reviewer, overall agreement on rating the morphological features of lesions [kappa statistic (95% confidence interval): 0.48 (0.410 - 0.548)]. In particular, reviewers tended to agree most on whether a punctum was present and on the homogeneity of lesions (overall 84% and 70% agreement, respectively). The color was agreed upon for 64% and border morphology was agreed upon for 61% of lesions. The lowest agreement occurred in rating the shapes (52%) and patterns of lesions (55%).

Final feature ratings for each lesion category are displayed in Table 2. Reviewer-classified EM lesions tended to be more oval than round, whereas the other lesion types tended to be more round. EM-classified lesions were also rated more often as pink, compared to the other classified lesion types that were more frequently rated as red, purple, or a combination of red, pink, or purple. Only two (6%) EM lesions had a ring-within-a-ring, or bull’s eye, pattern. A uniform pattern was the most frequent pattern observed for lesions classified in the EM category (51% of 35 lesions). All lesion types, including the EM-classified lesions, tended to have well-demarcated borders. Lesions classified as not-EM lesions, however, also commonly had irregular or raised borders (Table 2). EM, early EM, and tick bite reaction-classified lesions were most likely to be found on the torso, whereas none of the lesions classified as not-EM appeared on the torso (Table 3).

**Table 2:** Features of lesions within each reviewer-classified category Values are n (%) within each lesion category *Shape of one EM lesion could not be determined from the image ^†^Includes lesions that were brown or more than one color: red-purple, red-yellow ^‡^Patterns of two EM and one tick bite reaction lesions could not be determined from images ^§^Included geographic, blotchy, red ring just around punctum or papule ^ǁ^Border of one tick bite reaction could not be determined from images; also, three participants had lesions with more than one type of border: one in EM, one in tick bite, and one in not-EM, such that the denominators in the columns were 36, 7, 17, 12, and 72 for EM through total, respectively EM: erythema migrans

Lesion feature	EM (n = 35)	Suspected early EM (n = 7)	Tick bite reaction (n = 16)	Not-EM (n = 11)	Total (n = 69)
Shape*					
Round	12 (34)	4 (57)	10 (62)	6 (55)	32 (46)
Oval	22 (63)	2 (29)	2 (12)	2 (18)	28 (41)
Irregular	0	1 (14)	4 (25)	3 (27)	8 (12)
Color					
Red	7 (20)	4 (57)	8 (50)	3 (27)	22 (32)
Pink	26 (74)	1 (14)	7 (44)	4 (36)	38 (55)
Purple	1 (3)	1 (14)	0	2 (18)	4 (6)
Other^†^	1 (3)	1 (14)	1 (6)	2 (9)	5 (7)
Homogeneous	21 (60)	4 (57)	6 (37)	6 (55)	37 (54)
Pattern^‡^					
Ring-within-a-ring	2 (6)	0	0	1 (9)	3 (4)
Central lightness	4 (11)	2 (29)	3 (19)	1 (9)	10 (14)
Central darkness	7 (20)	0	1 (6)	2 (18)	10 (14)
Uniform	18 (51)	4 (57)	4 (25)	5 (45)	31 (45)
Other^§^	2 (6)	1 (14)	7 (44)	2 (18)	12 (17)
Border^ǁ^					
Well-demarcated	33 (92)	6 (86)	12 (71)	5 (42)	56 (78)
Raised/wheal	2 (6)	0	1 (6)	3 (25)	6 (8)
Irregular	1 (3)	1 (14)	3 (18)	4 (33)	9 (12)
Punctum present	11 (31)	7 (100)	16 (100)	1 (9)	35 (51)

**Table 3:** Frequencies of reviewer-classified lesions found at different body locations Values are n (%) of lesions within each category. Total unique locations for lesions were used as the denominators: 45, 7, 17, 11, and 80 for erythema migrans, suspected early erythema migrans, tick bite reaction, other, and total, respectively EM: erythema migrans

Location of lesion	EM	Suspected early EM	Tick bite reaction	Not-EM	Total
Upper body					
Neck	1 (2)				1 (1)
Shoulder			1 (6)	1 (9)	2 (2)
Torso					
Chest	2 (4)		1 (6)		3 (4)
Abdomen	10 (22)	2 (29)	4 (23)		16 (20)
Back	7 (16)	1 (14)	1 (6)		9 (11)
Hip	6 (13)				6 (7)
Upper extremities					
Armpit	2 (4)	1 (14)	1 (6)		4 (5)
Inner biceps	2 (4)	1 (14)	1 (6)	2 (18)	6 (7)
Arm	3 (7)		1 (6)	2 (18)	6 (7)
Elbow				2 (18)	2 (2)
Lower extremities					
Thigh	9 (20)		3 (18)		12 (15)
Knee	1 (2)		3 (18)		4 (5)
Calf	1 (2)	1 (14)		1 (9)	3 (4)
Shin		1 (14)		2 (18)	3 (4)
Ankle	1 (2)			1 (9)	2 (2)

Participants whose lesions were classified as EM reported that they had the rash or LD symptoms for more days prior to enrollment compared to participants within the other categories (average number of days: 7.5 for the EM group versus 3.9 days for all other groups; p = 0.043). Although one-third of the participants with reviewer-classified EM lesions reported no other symptoms, two-thirds (66%) reported at least one symptom compared to only 47% of participants in the other groups (Table 4). Participants with reviewer-classified EM lesions also had a higher number of symptoms (average of four versus two symptoms, p = 0.017). Compared to other categories, participants with reviewer-classified EM and tick bite reaction lesions were significantly more likely to report headaches, body aches, and nausea (p: <0.05; Table 4). Sex and age were not significantly associated with the type of reviewer-classified lesions (Table 1, p = 0.20 and 0.078, respectively).

**Table 4:** Summary of symptoms reported by participants within each reviewer-classified lesion category Values are n (%) within lesion categories *Fisher’s exact test EM: erythema migrans

Symptom present	EM (n = 35)	Suspected early EM (n = 7)	Tick bite reaction (n = 16)	Not-EM (n = 11)	Total (n = 69)	P-value*
None	12 (34)	7 (100)	4 (36)	7 (44)	30 (44)	
At least one	23 (66)	0	7 (64)	9 (56)	39 (56)	
Median (max) number of symptoms	4 (10)	0	2 (9)	1 (5)	1 (10)	
Fever	10 (29)	0	5 (31)	2 (18)	17 (25)	0.423
Chills	15 (43)	0	5 (31)	2 (18)	22 (32)	0.105
Night sweats	16 (46)	0	4 (25)	2 (18)	22 (32)	0.060
Fatigue	18 (51)	0	7 (44)	5 (45)	30 (43)	0.086
Headache	16 (46)	0	7 (44)	0	23 (33)	0.003
Body aches	19 (54)	0	9 (56)	2 (18)	30 (43)	0.009
Neuralgia	9 (26)	0	3 (19)	1 (9)	13 (19)	0.468
Nausea	13 (37)	0	3 (19)	0	16 (23)	0.023

Of the 35 participants with reviewer-classified EM lesions in this study, 12 (37%) had positive serologic evidence and one had molecular evidence for *B. burgdorferi* infection with negative Lyme serology at the acute phase (Table 5). Laboratory evidence for LD was also detected in one participant whose lesion was classified as a tick bite reaction; this individual was also the only participant with evidence of seroconversion (Table 5). Positive serologic evidence was not significantly related to participant demographics or other clinical characteristics (all p: ≥0.276).

**Table 5:** Laboratory test results at acute- and convalescent-phase blood draws within each of the reviewer-classified lesion categories Values are n (%) within lesion categories *Not all participants donated a convalescent-phase blood sample ^†^If only the IgM immunoblot was positive in the convalescent sample, this was not considered a positive STTT result because the draw was >30 days from the onset of illness [25] ^‡^Seroconversion was defined as a positive IgG immunoblot at the convalescent-phase visit, following a negative STTT at the acute-phase visit EM: erythema migrans; PCR: polymerase chain reaction; STTT: standard two-tier testing

Testing result	EM	Suspected early EM	Tick bite reaction	Other	Total
Acute-phase visit					
N	35	7	16	11	69
PCR-positive	1 (3)				1 (1)
Standard two-tier-positive	12 (34)				12 (17)
Positive on first-tier only (VlsE/pep10 and/or C6 ELISA)	7 (20)	1 (14)	5 (31)	5 (45)	18 (26)
Negative on all serologic tests	14 (40)	6 (86)	10 (62)	6 (56)	36 (52)
Convalescent-phase visit*					
N	24	7	14	7	52
Standard two-tier-positive^†^	1 (4)				1 (2)
Positive on first-tier only (VlsE/pep10 and/or C6 ELISA)	15 (62)	1 (14)	2 (14)	3 (43)	21 (40)
Seroconversion^‡^			1 (7)		1 (2)
Negative on all tests	8 (32)	6 (86)	11 (79)	4 (57)	29 (56)

## Discussion

Early LD diagnosis is known to be challenging and is largely dependent on physical findings and recognition of EM [14,18]. Upon their first assessments, our clinician reviewers assigned 61% of the 69 lesions evaluated in our study to the same categories, suggesting that experienced providers in different fields of medicine, practicing in different LD-endemic regions can have a moderate agreement in recognizing EM. However, the fact that they did not find higher levels of agreement emphasizes the challenges. The moderate agreement found in the inter-reviewer ratings of the physical features of the lesions points to the variation that can exist and the element of subjectivity in interpreting the morphology of the lesions [14,17,18,27]. Significantly, we only observed two lesions classified as EM (6% of 35) that exhibited a ring-within-a-ring, or the classic bull’s eye, target pattern often used to describe EM lesions. The most frequently observed type of EM was the uniform, homogeneous pattern (49% of 35), followed by lesions with darker centers (20%), and lesions with central lightness or clearing (14%) (Table 2). Earlier descriptions of EM in Wisconsin and studies in Northeastern states have also reported more commonly encountering homogeneous, expanding lesions, rather than lesions with central clearing [7,15,28]. Even the initial descriptions of EM by Steere et al. [12,29] included lesions that remained homogeneous as they expanded. This general finding across studies suggests that education should deemphasize the bull’s eye form and stress the wide variability in EM instead and the fact that many of them present as a uniform, homogeneous lesion.

Differentiating between EM, possible early EM, and tick bite reactions was the primary challenge for our reviewers. Of the initial 27 discordant lesions, 18 (67%) were lesions that reviewers disagreed on as to which of these three categories they belonged. The morphological features of these categories overlapped, and the interpretations of lesion colors, shapes, and patterns, in general, could be subjective. In particular, there were sometimes slight differences between round and oval, pink and red, and in uniform patterned lesions versus lesions with slight central clearing or darkness. Final classification suggested EM lesions tended to be larger in size, more oval than round in shape, and were more pink than red, compared to the early EM or tick bite lesions, which were rounder and darker in color. Additionally, EM lesions were less frequently observed to have a distinct punctum present, whereas a punctum was common in early EM and tick bite reaction lesions (Table 2). It is unclear how generalizable such morphologic features may be; however, Rebman et al. [27] have reported higher frequencies of red and round EM lesions in a cohort of LD patients recruited in Maryland and southeastern Pennsylvania. It has been suggested that the shape and color of EM may relate to the duration of the localized reaction to the tick bite or spirochetes, the location of the body, and orientation relative to lines of skin tension [15,27].

One-third of the lesions in our study (23/69) were ultimately classified into the possible early EM or tick bite reactions categories, suggesting that patients commonly seek care very early in the course of a possible LD illness with an EM. This places an additional burden on providers in highly endemic areas to make clinical decisions before all information may be available and when laboratory diagnostics perform poorly [9,10]. As such, LD diagnosis and decisions to treat will depend on the clinician's experience and tolerance for uncertainty. In our study, 39% of the 23 participants classified into the early EM or tick bite reaction groups reported at least one LD symptom and one of the 16 (6%) lesions identified as tick bite reactions ultimately had confirmatory laboratory evidence of LD. Additionally, 87% of these participants reported a tick bite, and 52% presented during peak tick season (May-July), suggesting that participants were generally being correctly evaluated for LD by their clinicians. When presented with a lesion that may be a tick bite reaction or an early EM, providers have the option of starting the treatment or waiting for the lesion to expand or for additional LD symptoms to develop. Given the difficulty of distinguishing between a tick bite reaction and an EM, and the costs of secondary LD sequelae, if not treated early, the decision to treat for LD in cases of uncertainty in highly endemic areas may be appropriate. Indeed, decision models to evaluate the costs and outcomes related to empirically treating or not treating tick bites or EM-like lesions for LD in endemic areas support the decision to treat [30,31].

Similarities between EM and skin lesions of other etiologies can lead to misdiagnoses. In particular, EM may be confused for cellulitis, spider bites, poison ivy, and urticaria, and, conversely, these other lesions may be misdiagnosed for EM [17,18,32]. There were also lesions observed in our participants whose clinicians suspected early LD and who our reviewers identified with other possible diagnoses, including ringworm, solar purpura, and contact dermatitis (Figure 4). Though distinct morphological features of EM were not evident from our evaluations, a few general differences between EM and lesions classified as not-EM were observed. EM lesions rarely had irregular borders, as was observed in some of the not-EM lesions, including contact dermatitis (Figure 4B), pruritus associated with a tick or other arthropod bite (Figure 3F), and dermatitis caused by a bandage applied to a bite (Figure 3E). Solar purpura had a geographic pattern not seen in EM (Figure 4C), and scaling was observed in ringworm, which may resemble a bull’s eye (Figure 4A), but is not seen in EM.

Limitations of our study included not having confirmatory laboratory evidence of *B. burgdorferi* infection for the majority of our cohort. Consistent with the known low sensitivity of diagnostic testing during early LD [9,10,33], only 37% of participants in the EM category had laboratory evidence of LD. Direct detection methods for* B. burgdorferi* include the isolation of spirochetes and their molecular detection from a skin biopsy sample taken from the EM lesion or blood. However, the methods are generally insensitive and are not routinely employed for LD diagnosis [33]. Another possible limitation was the moderate agreement observed between our reviewers. Variations in the quality of the exposure and framing of some of the lesions in the images and the lack of clinical information accompanying the images may have contributed to the moderate agreement. A higher level of agreement may have been achieved if the study had been designed to have our reviewers examine lesions in person, with access to patient history and physical information. However, the aim of our study was to try to identify morphological features associated with EM, unbiased by other information. As discussed, there is also inherent variability in the features that may be subject to different interpretations, such as shape and color. Finally, our cohort only included White, non-Hispanic participants, who were primarily middle-aged, and hence the morphological trends we observed may not be generalizable to EM in other races or children. Failure to observe more lesions with central clearing, which has been reported to be associated with younger patients [27], for instance, may have been due to few children being included in our study. Moreover, the ring-within-ring, or the bull’s eye form may be associated with a longer duration of EM [12]. That the majority of the lesions in our study were of short duration (54% were present for seven days or fewer) may explain the low frequency of such lesions. However, given that patients may commonly present early in the disease course, healthcare providers should be made aware that ring-within-ring lesions may be encountered less frequently.

## Conclusions

Our findings corroborate other reports stating that EM commonly presents as a homogeneous, uniform lesion that may have central darkness or clearing, but infrequently presents with a bull’s eye, or ring-within-ring, pattern. The lesions tend to be pink-to-red, oval-to-round, with well-demarcated borders and no punctum. Though participants with reviewer-classified EM lesions were more likely to have lesions for a longer duration, present with early LD symptoms, and have laboratory evidence for LD, 34% and 51% of these participants did not have any other symptoms or failed to have confirmatory laboratory evidence for LD, respectively. Therefore, clinical knowledge incorporating other aspects of the disease, such as its endemicity and seasonality, and an understanding of an individual patient’s risks of tick exposure, are needed to make the clinical diagnosis. Moreover, providers should be aware that many patients in LD-endemic areas will present either in the very early stage of LD or with a tick bite reaction, and that they may need to decide to treat LD with incomplete patient information and guidelines. Further efforts are needed to measure the knowledge gaps of healthcare providers and to improve the recognition of EM to help them in their clinical decision-making.
